# Comparing Temporospatial Performance During Brisk and Self-Paced Walking by Men With Osteomyoplastic Transfemoral Amputation and Controls Using Pressure and Muscle Activation Peak Times

**DOI:** 10.3389/fresc.2022.848657

**Published:** 2022-05-06

**Authors:** Monique O. Shotande, Kimberly P. Veirs, Jonathan D. Day, William J. J. Ertl, Andrew H. Fagg, Carol P. Dionne

**Affiliations:** ^1^School of Computer Science, University of Oklahoma, Norman, OK, United States; ^2^Department of Physical Therapy, Oklahoma City University, Oklahoma City, OK, United States; ^3^Department of Orthopedic Surgery and Rehabilitation, University of Oklahoma Health Sciences Center, Oklahoma City, OK, United States; ^4^Institute for Biomedical Engineering, Science and Technology, University of Oklahoma, Norman, OK, United States; ^5^Department of Rehabilitation Sciences, University of Oklahoma Health Sciences Center, Oklahoma City, OK, United States

**Keywords:** transfemoral osteomyoplastic amputation, gait cycle, residuum socket pressures/forces, electromyography (EMG), co-contraction

## Abstract

**Background:**

The aim of osteomyoplastic transfemoral amputation (OTFA) is to produce sustained, robust prosthetic gait performance by residuum reconstructing. A better understanding of residuum-socket interface pressures (RSI) and residuum muscle activation should uniquely reveal gait stability to better inform long-term rehabilitation goals.

**Objectives:**

The objectives of this study are to characterize RSI pressures and residuum muscle activation in men with OTFA while walking at two speeds and compare temporospatial muscle activation with intact controls.

**Methods:**

In this study, we observed and compared healthy men with OTFA and controls during 2-min gait trials at brisk and self-paced speeds, two visits, and 1 year apart. RSI pressures and hip adductors, hamstrings, and quadriceps activation were recorded for those with OTFA. OTFA temporospatial muscle activation patterns were compared with the controls. Within the extracted strides, heel-strike and toe-off events and EMG activation peak times were characterized and compared. Peak times for pressure and EMG activity were examined in individual muscles and antagonist muscles of residual and intact limbs.

**Results:**

Six men with OTFA exhibited adductor, hamstring, and quadriceps co-contraction within intact and residual limbs, regardless of walking speed or trial. Co-contraction within their intact limb occurred throughout the gait cycle. Within the residuum, co-contraction occurred during weight transference. The 75% most likely RSI peaks occurred during stance. EMG peaks were 75% most likely to occur during early stance, terminal stance-initial swing, and terminal swing.

**Conclusion:**

Participants with OTFA demonstrated adductors-hamstrings-quadriceps co-contraction in the intact thigh and residuum with corresponding RSI pressure increase, primarily during transitions between stance and swing, indicating gait instability, demonstrating the need to explicitly address these deficits continuously in rehabilitation and wellness settings.

## Introduction

Despite advances in prosthetic management and surgical approach, healthy individuals with osteomyoplastic transfemoral amputation (OTFA) still risk continued impairment and disability (1). Stable gait performance within the community is key to overall health and participation. However, frequent missteps and falls often result in painful residuum injury, resulting in diminished participation by this vulnerable group (2). Little is known about the roles the distal-most residuum muscles, or the pressures produced at the RSI play during gait performance. Clinicians treating patients with OTFA in rehabilitation and wellness settings need to more fully understand the roles of the distal-most muscles and RSI pressures during active prosthesis use for optimal outcome.

In those with transtibial amputation (TTA), there is evidence of active contribution to prosthesis control during gait by the distal-most muscles (i.e., anterior tibialis, gastrocnemius), specifically in adult men with osteomyoplastic TTA (OTTA) (3). However, little is known about the distal-residuum muscle activity or RSI pressures produced during brisk or self-paced gait performance by individuals with OTFA (4). This information may be key to better prepare patients with OTFA for more successful, in-community participation. Most current rehabilitative approaches after OTFA focus on gait training, joint mobility, and proximal muscle strengthening (5). However, insufficient attention has been directed at (1) OTFA residuum muscles distal to the hip (i.e., adductors, hamstrings, and quadriceps); and (2) pressures produced at the RSI during prosthetic gait (6).

A growing number of adults with OTFA have undergone this innovative type of surgical amputation. The intent of the osteomyoplastic procedure is to yield a viable, reconstructed residuum for weight-bearing and reliable prosthetic fit and use. Active gait performance is the primary, expected outcome (7). Mai et al. (3) documented novel muscle activity in the distal OTTA residuum, and Dionne et al. (8, 9) found comparable work performance (gait, lifting, and carrying) in these same male participants with OTTA, as intact controls. Thus, investigators expected that men with OTFA would perform better or similar to controls, despite missing a knee joint system, shank, ankle joint system, and foot (4).

However, Dionne et al. (1) found that work performance by men with trauma-related OTFA fell short of their intact, matched cohorts in all tasks tested (i.e., lifting, carrying, self-paced, and brisk walking). Each participant with OTFA presented with a well-fitted prosthesis (1, 10) (submitted) but reported only receiving standard, short-term rehabilitation. Therefore, in this study, we focused on characterizing the RSI pressures and residuum muscle activity during independent gait performance to determine gait stability for the long-term in this unique cohort.

It is paramount that individuals with OTFA following amputation are safely able to achieve and maintain gait stability once returned to the community. For this purpose, lower limb prosthetics are designed to facilitate efficient biomechanical performance during walking. Proper fit of the prosthetic socket and the dynamics of the prosthetic components are key for patients to obtain similar gait performance to that before amputation. Improper design can result in pathological gait patterns that nefariously impact the individual's overall health and well-being and can subsequently result in developing health complications in otherwise healthy individuals.

Presently, it is not well understood in OTFA how- or when- the osteomyoplastic residuum muscles activate during gait tasks at variable speeds, specifically by the adductors, hamstrings, and quadriceps, in relation to the pressures exerted at the RSI (4). Wentink et al. (11) found less consistency in TFA walking patterns. However, the amputation approach of the participants was not specified, additionally, the TFA cohort was not homogeneous, as there were broad inclusion criteria and the prosthetic fit was not verified, making it difficult to isolate effects due to the amputation approach. Additionally, hip muscles of the intact limb were not measured. Another study by Jaegers et al. (12) discovered longer activation times for hip muscles within intact and residual limbs of individuals with TFA. However, a limitation of prior studies of TFA is the small sample size of strides per subject. Moreover, the co-activity of muscles is assessed only after averaging EMG activity across strides, as opposed to assessing co-activity on a per-stride basis.

In this paper, we examined distal-residuum muscle activity and RSI pressures during brisk and self-paced gait performed by healthy men with OTFA and compared their performance with that of intact controls. We show that RSI pressures are distributed throughout the residuum-socket and that muscles are engaged during gait and are often co-contracted at key times within the gait cycle. These results have potential implications for improving rehabilitation outcomes and reducing the risk of injury.

## Methods

This is a cross-sectional observational study approved by the institutional review board. Participants were recruited from a regional Trauma I Center in the central United States. Selection criteria were as follows: formally consenting men aged 18–64 years who were otherwise healthy, had no other interfering chronic condition or need for an assistive device, and underwent OTFA at least 6 months prior to the study. One individual with OTFA underwent knee disarticulation.

### Protocol

The study protocol is summarized in Supplementary Figure S2 (1). All participants engaged in gait trials at two speeds, during two visits, 1 year apart. The order of the walking speed trial was based on a coin flip. Once sensors were attached and tested, participants walked at one pace for 2 min, rested, and then walked for 2 min at the other speed. Vital signs and reports of perceived exertion were recorded to ensure participant safety (13). Pressure and muscle activation data were collected using the OU-PAM (3, 14), which can capture 16 analog input channels at 1 kHz. It utilizes the STK525 (15) and ATEVK525 (15) boards for data acquisition. Similar sensor locations are used here except for two residuum EMG sensors moved to the adductors and hamstrings for the OTFA cohort (Supplementary Figure S1).

#### Pressure

For control participants, pressure sensors were placed on the heel and ball of each foot to detect heel-strike and toe-off events, respectively. Participants with OTFA had eight pressure sensors placed within the prosthetic socket at the distal (D) and proximal (P) levels of the remnant femur (Supplementary Figure S1). Proximal sensors were placed at the 1/3-femur length level, measured downward from the ischial-level within the socket. Four sensors were placed at each level, at the anterior (A), posterior (P), lateral (L), and medial (M) positions. Heel-strike and toe-off for each respective limb was extracted from each trial referencing the average RSI pressure. Pressures for the intact limb were not instrumented by the OU-PAM, therefore, stance start and end times were inferred from unloading and loading events with the residuum-prosthetic limb. The time the average RSI pressure begins to peak is designated as the stance start by the residual limb. The time the same peak attenuates is the stance end. Strides are then extracted as heel-strike to heel-strike with the same limb, with time duration within the 2–95th percentile range. This yielded 67–126 strides per participant per task. Complete detail of the extraction procedure is described by Shotande et al. (10).

#### Muscle Activation

Surface EMG sensors were placed over the adductors, hamstrings, and quadriceps muscle group bellies on the residual and intact limbs (Supplementary Figure S1). For each stride, peak EMG activation times were determined to characterize muscle recruitment strategies and gait performance. Local EMG maxima within a sliding window and above the 50th percentile activation were selected as points of peak activation (10).

### Data Analysis

Descriptive data were analyzed for participant comparability (1). Six of 20 control participants that matched the age, height, and weight of the six participants with OTFA were selected for analysis. Individual participant muscle activation and pressure data were collected and one visit was selected for analysis (10). Some participants did not complete a second visit; for participants with two visits, the one with the most strides and fewest data artifacts was chosen. Pressure and EMG data were normalized and filtered using low-pass first-order Butterworth filters, based on the approach outlined by Commuri et al. (14) and Shotande et al. (10).

Peak activation was considered part of a mobilization and control strategy. Therefore, the analysis consisted of obtaining the peak time distributions for pressure and EMG activation within a stride. Peak times were aggregated across the participants for each pressure location and muscle group. Areas of high-density peak times indicate consistent periods during the gait cycle of high relative pressure or muscle activity. Moreover, EMG peak times for the three muscle groups were paired within the stride to evaluate co-contraction.

### Statistical Analysis

Two-tailed, two-sample *t*-tests, with α = 0.05, were performed to assess the comparability of the demographic information (i.e., age, height, and weight) between subject groups. Two-sample Kolmogorov-Smirnov (KS) tests were conducted for each muscle and RSI location to test the null hypothesis of whether the peak times for brisk and self-paced walking are from a distribution with the same shape. The α = 0.05 was Šidák-corrected to account for multiple comparisons (16). Highest density regions (HDRs) (17) were computed for 95% and 75% accumulated probabilities to compare specific time periods between participant groups, muscle groups, and walking speeds. HDRs are the shortest intervals, consisting of the most likely peak times, whose likelihoods integrate to (1−α_*i*_). The values α_1_ = 0.05 and α_2_ = 0.25 were the significance levels used to determine the threshold likelihood for the 95% and 75% HDRs, respectively. Time points with likelihoods ≥*f*_α_1__ were within the 95% HDR. Time points with likelihoods ≥*f*_α_2__ were within the 75% HDR.

## Results

### Participants

Six participants with OTFA and six controls meeting the selection criteria completed the study. No participants reported any significant increase in pain *via* Visual Analog Pain-Rating Scale (0.5/10[±1.2]) or significant changes in reports of perceived exertion (13) (brisk-pace RPE=10.5[range: 8–13]; self-pace RPE=8.5[range: 6–11]) during the trials (1). Data of the matched controls by age, height, and weight, with valid data for all sensors and walking tasks, were aggregated to compare with the OTFA group. There was no significant difference in age (*p*>0.3), height (*p*>0.3), or weight (*p*>0.7) between the participants with OTFA and the matched controls. The average age of the OTFA cohort and the matched controls was 34.0±15.0*yrs* and 27.2±6.2*yrs*, respectively. The average height for both cohorts was 1.8±0.1*m*. The average weight for the OTFA cohort and the matched controls was 83.3±16.4 and 87.3±20.0*kg*, respectively.

### Distributions of Peak Times

#### RSI Pressures

##### Participants With OTFA

**Figure 1:**
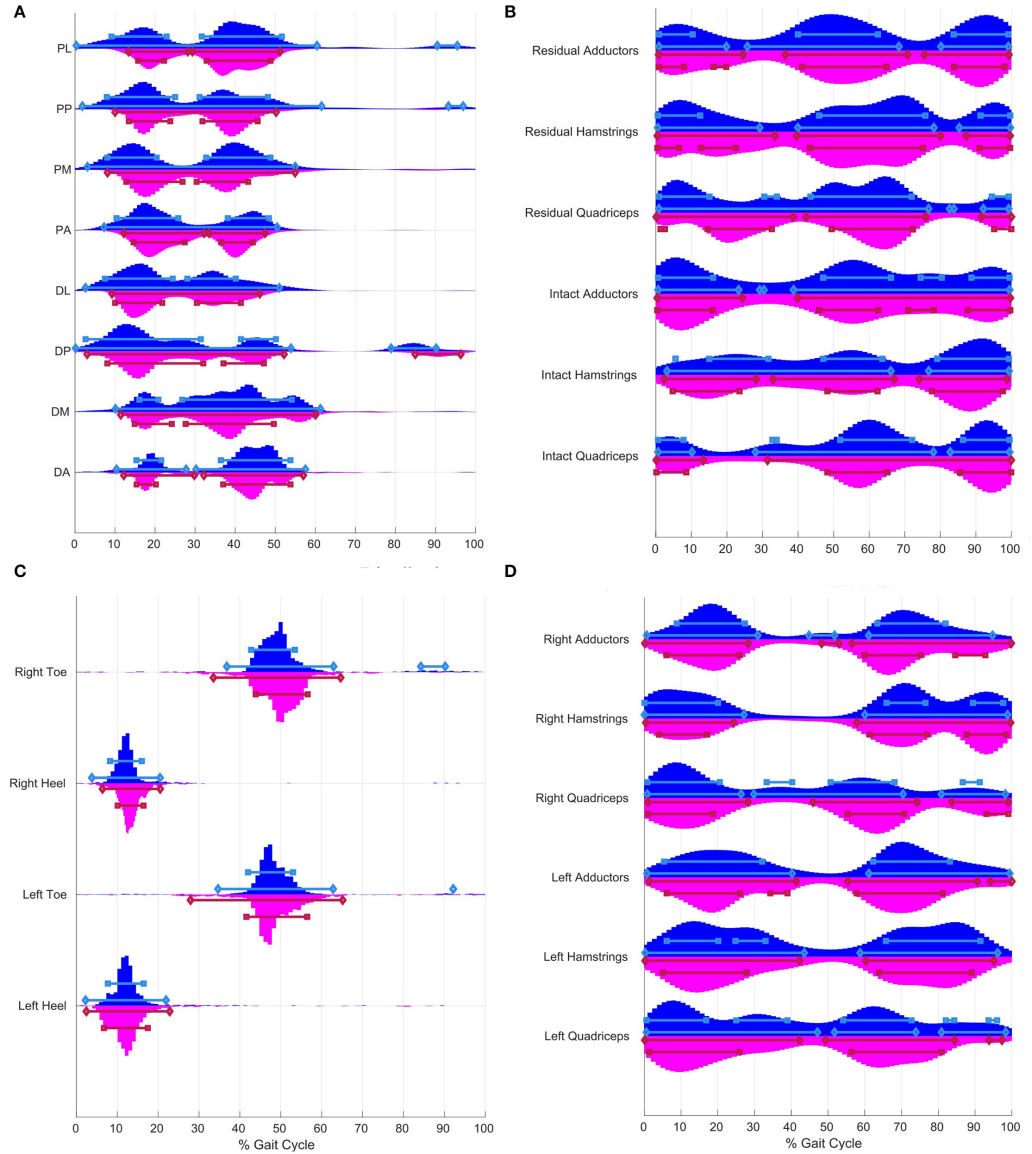
Distribution of peak times for residuumsocket interface (RSI) pressure **(A)** and electromyography (EMG) **(B)** from an osteomyoplastic transfemoral amputation (OTFA) cohort of 6 and foot pressure **(C)** and EMG **(D)** from 6 matched controls. Horizontal bars indicate the highest density regions (HDRs) with accumulated probabilities of 95% (diamond endpoints) and 75% (square endpoints) for brisk (blue) and selfpaced (red) walking, respectively. **(A)** Eight RSI pressure sensors are placed along two levels, proximal (P) and distal (D), at the anterior (A), posterior (P), lateral (L), and medial (M) positions, also (see Supplementary Figure S1A). Over 95% of RSI pressure peaks occur prior to 60%. Prior to 60% of the gait cycle, there are two time segments at each RSI location, designated by 75% HDRs. The 95% HDRs for selfpaced are shorter than for brisk walking and start later. **(C)** Foot pressure sensors are on the heel and ball of the foot. Over 95% of peaks at the heel occur 0–25%. Approximately, 95% of toe pressure peaks occur around pushoff (i.e., 27.9–65.2%) and during terminal swing (i.e., 92.1–92.2% and 84.2–90.2%). The 95% HDRs for selfpaced are longer than for brisk walking, and start earlier and end later. **(D)** The controls have two major HDRs for each muscle group, at approximately 0–30% and 50–100%. The third region for hamstrings and quadriceps during the terminal swing, approximately 80–100%. Violin plots were constructed using code based on functions by Jonas (18).

The distributions of peak RSI pressure times for all valid strides (≥67 per participant) for six individuals with OTFA at brisk (blue) and self-paced (magenta) walking speeds are shown in Figure 1. The HDRs with accumulated probabilities of 95% (diamond endpoints) and 75% (square endpoints) are depicted with horizontal bars drawn above the line corresponding to the respective sensor. These distributions are multi-modal, and HDRs can be comprised of multiple time segments. The probability that peaks occur within one of these time segments is 95% for the 95% HDRs. The 95 and 75% HDRs of RSI pressure peaks are prior to 61% of the gait cycle, at all RSI locations. Additionally, RSI pressure peaks are likely to occur between 90.5 and 95.5% at the PL location for brisk walking, 93.3–96.9% at the PP location for brisk walking, 78.9–90.2% at the DP location for brisk walking, and 85.0–96.3% at the DP location for self-paced walking. The 95% HDRs are longer for brisk walking than self-paced, at all RSI locations. These time periods start later in the gait cycle for self-paced, than brisk walking, and end earlier for self-paced. During the stance phase there are two periods, indicated by the 75% HDRs, at each socket location, when peaks are most likely to occur. These periods correspond to weight acceptance (between 0–30%) and to push-off (between 30–60%). The period of time that peak pressures occur during early stance is shortest at the DM and DA locations, compared to the other locations. During push-off, the distribution of peak times at the DM location is the longest compared to other locations.

There are significant differences in the shape of the distributions of peak times between the two walking tasks, for the RSI locations with the exception of the DA (Supplementary Table S1A). Despite overlaps in the HDRs, there are differences in duration. Moreover, there are shifts in peak times where a corresponding region for one task might be earlier or later, compared to the other task. For example, at the PM location, the HDRs during early stance for self-paced walking start later than the regions for brisk walking.

##### Controls

The distributions of peak foot pressure times for all valid strides from the matched controls at both gait speeds are shown in Figure 1C. Approximately, 95% of pressure peaks at the heel occur between 2 and 22.9%. Approximately, 95% of pressure peaks at the toe occur between 27.9 and 65.2% during the gait cycle. Additional toe pressure peaks within the 95% HDRs for brisk walking occur between 84.2–90.2% for the right foot and 92.1–92.2% for the left foot.

There are significant differences in the shape of the distributions between the two walking speeds, for the foot pressures (Supplementary Table S2A). While the HDRs are similar, there are differences in duration and shifts in time where corresponding regions for one task might be earlier or later, compared to the other task. For instance, HDRs are longer relative to the normalized time for self-paced walking than for brisk walking, for the toe. Additionally, toe pressure peaks are likely to occur after 84% (e.g., 92.1–92.2% and 84.2–90.2%), only during brisk walking.

#### Muscle Activation

##### Participants With OTFA

The distributions of peak EMG activation times from the participants with OTFA, at both gait speeds are shown in Figure 1B. The 95% HDRs of peak times in the EMG activity are separated into two to three-time segments. For example, during brisk walking within the residual hamstrings, 95% of the most likely peaks occur approximately 0.9–28.2%, 39.3–78.8%, and 85.9–99.1%. For the intact hamstrings during brisk walking, the 95% most likely peak times are 0.9–65.7% and 77.8–99.1%. The 75% HDRs of peak times for the EMG activity are separated into three to four time segments. For all muscles and tasks, peaks are most likely to occur throughout the load response, pre-swing, and terminal swing. There are also shorter time segments only during mid-stance, such as for the residual adductors for self-paced (17.1–19.1%), or only during mid-swing, such as for the intact adductors for brisk (73.8–80.9%).

There are significant differences in the shape of the distributions between the two walking speeds, for the EMG muscle groups (Supplementary Table S1B). Despite similar patterns between tasks, the HDRs do not start and end at the same time. For instance, the 75% HDR during terminal stance for brisk walking, with the intact quadriceps, occurs earlier for self-paced than brisk walking.

##### Controls

The distributions of peak EMG activation times from the matched controls are shown in Figure 1D. The HDRs of peak times in the EMG activity occur during two time segments designated by two 95% HDRs for each muscle during both gait tasks. These segments are divided into sub-intervals by the 75% HDRs. During the early stance, the 95% HDRs for the right limb muscles are 0–30.9% and the 75% HDRs for the right limb muscles are 0–27.4%. During early stance, for the left limb, the 95% HDRs are longer, 0–47.1%, and the 75% HDRs are 0–39.0%, with the hamstrings and the quadriceps having two sub-regions in between. The 95% HDRs end later for the left than for the right limb. For the right quadriceps for brisk walking, peaks are also likely to occur between 29.9 and 71.1%. In the segment 50–100%, 95% HDRs either extend throughout half or more of the swing, and start either during pre-swing or initial swing, with exception of the right quadriceps for brisk walking, where a 95% HDR interval begins at 29.9% and extends into the swing, ending at 71.1%. Additionally, a short 95% HDR during terminal stance within the right adductors is between 44.8 and 51.8% for brisk and 48.2–52.9% for self-paced.

There are significant differences in the shape of the distributions between walking speeds, for the EMG muscle groups (Supplementary Table S2B). The HDRs differ in duration and some do not overlap between gait speed, such as for the right quadriceps during midstance between 30 and 40%. There are 95% and 75% HDRs for brisk, but not for self-paced.

### Co-Contraction

#### Participants With OTFA

**Figure 2:**
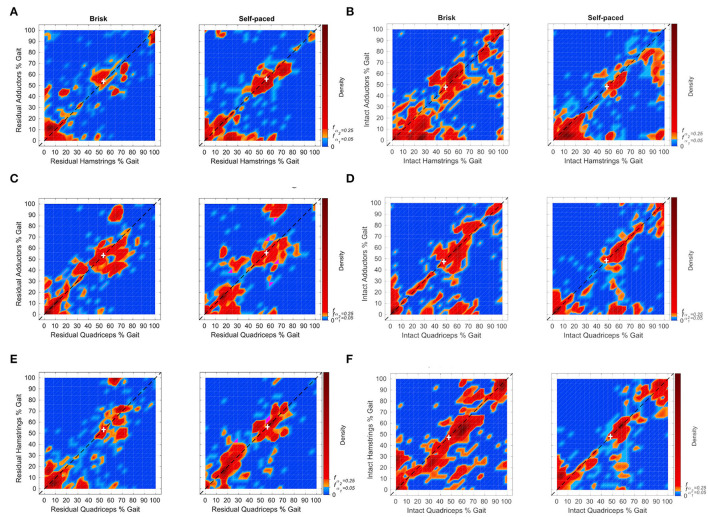
Distributions of muscle peak pairing times between the adductors, hamstrings, and quadriceps muscle groups, from 6 individuals with OTFA for the residual **(A,C,E)** and intact **(B,D,F)** limbs. Individual points can be found in Supplementary Figure S6. Time zero corresponds to the heel-strike of the corresponding limb. Pairings that occur near or along the diagonal indicate co-contraction between muscle groups. The white cross indicates the mode of the distribution of the stance duration. Distributions of the stance duration are in shown Supplementary Figure S3. The subfigures depict the relative frequency of peak pairing times that occur during the gait cycle. Orange and red are within the 95% high density region (HDR). Red indicates points within the 75% HDR. Magenta refers to points with density equal to the 95% HDR threshold f_α1_. For the residual limb, HDRs along the diagonal are prior to 80%, with the exception of residual adductorshamstrings for both tasks and adductorsquadriceps for selfpaced. The HDR after 90% for residual adductors-hamstrings for brisk is composed of muscle peak pairings largely from one participant. The HDR after 90% for residual adductors-hamstrings for self-paced is composed of peak pairings from two subjects. The HDR after 90% for residual adductorsquadriceps for self-paced is composed of peak pairings from three subjects. For the intact limb, HDRs along the diagonal occur throughout the gait cycle.

The density of peak times between pairs of residuum muscles from all OTFA participants for brisk (left) and self-paced (right) walking are shown in Figures 2A,C,E (peak times for individual strides are shown in Supplementary Figure S6). The HDRs for 75% (red) and 95% (orange and red) are the most likely time periods for peak activation between muscles to be matched. Regions shaded in magenta have likelihoods equal to the 95% HDR threshold, *f*_α_1__. The HDRs near or along the diagonal are the most likely co-contraction times, and occur prior to 80% of the gait cycle, with sub-regions between 0–30% and 40–80%. The off-diagonal HDRs are not co-contraction and contribute to momentum, not stability. The white cross indicates the mode of the distribution of the stance duration (Supplementary Figure S3). For residual adductors-hamstrings, co-contraction at both speeds (Figure 2A) and the residual adductors-quadriceps co-contraction at self-paced (Figure 2C), there is also an HDR on the diagonal after 90%. The density of co-contraction times for the intact limb muscles are shown in Figures 2B,D,F. The HDRs near or along the diagonal extends throughout the entire gait cycle, with small gaps (less than 10%).

#### Matched Controls

**Figure 3:**
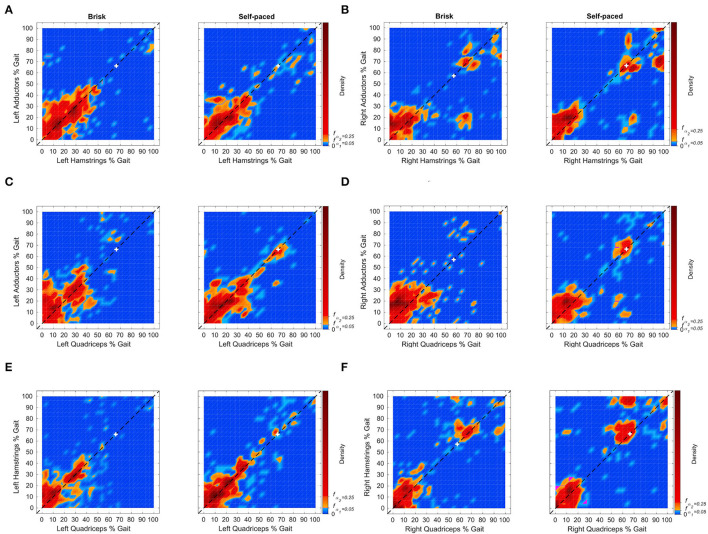
Distributions of muscle peak pairing times between adductors, hamstrings, and quadriceps muscle groups from 6 matched controls for the left **(A,C,E)** and right **(B,D,F)** limbs. Individual points can be found in Supplementary Figure S7. Time zero corresponds to the heel-strike of the corresponding limb. Pairings that occur near or along the diagonal indicates co-contraction between muscle groups. The white cross indicates the mode of the distribution of the stance duration. Distributions of the stance duration are shown in Supplementary Figure S3. The subfigures depict the densities of peak pairings during the gait cycle. Orange and red indicate periods within the 95% HDR. Red indicates points within the 75% HDR. Magenta refers to time points with density equal to the 95% HDR threshold, f_α1_. For the left limb, large HDRs for co-contraction are prior to 60%, for all muscle pairs. Small HDRs also occur during the swing for selfpaced walking between the adductor-shamstrings, adductor-squadriceps, and hamstring-squadriceps and during brisk walking between the adductor-shamstrings and adductor-squadriceps. For the right limb, large HDRs for co-contraction are prior to 40%, for all muscle pairs. HDRs of equal or lesser area occur after 60%.

For the matched controls, density of peak activation times between pairs of muscles for each limb is shown in Figure 3. The individual data points in the distributions can be seen in Supplementary Figure S7. Large HDRs for co-contraction are during stance, prior to 60%, for all muscle pairs within the left (Figures 3A,C,E) and right (Figures 3B,D,F) limbs of the matched controls. For the right limb, HDRs prior to 60% are shorter than those within the left limb, between 0 and 35%. There are additional periods within the 95 and 75% HDRs after 60%, within the right limb. Small HDRs also occur during the swing for self-paced walking within the left limb for adductors-hamstrings, adductors-quadriceps, and hamstrings-quadriceps pairings and during brisk walking for adductors-hamstrings pairings.

## Discussion

### Pressure

The RSI pressure peak time distributions (Figure 1A) have similar patterns to the foot pressure distributions of the controls (Figure 1C). A set of HDRs for the RSIs span prior to 30% of the gait cycle. This is comparable to the HDRs for the heel-strike of the controls, also spanning prior to 30%. However, the HDRs for the heel pressures of the controls are narrower. The second set of HDRs after 30% is comparable to the toe pressure distributions of the controls. However, the HDRs for the toes end later than those of the RSIs. Moreover, RSI pressure peak distributions mimic the pattern of vertical ground reaction forces as observed by Alamdari and Krovi (19). RSI pressures peak most often during residual limb weight-bearing and push off. The set of sensors shows similar timing of peak pressure to one-another, indicating distributed spatial contact with the prosthetic socket, therefore, a good prosthetic fit.

### Muscle Activation

Increased rates of adductor-hamstrings-quadriceps co-contraction were observed throughout the gait cycle within the intact limb and at times of residuum weight transference. In Figure 2, the 95 and 75% HDRs are seen for each muscle pairs for the OTFA cohort. HDRs for muscle peaks near or along the diagonal indicate co-contraction. All other peaks offset in time likely contribute to momentum and not stability. The HDRs of co-contraction for the intact limb occur during the entire gait cycle and have a high variance of peak time, suggesting increased reliance on the intact limb for stability during all gait phases, regardless of pace (Figures 2B,D,F). The HDRs of co-contraction for the residuum are primarily during weight acceptance and push-off (Figures 2A,C,E). All three recorded muscles are activated at initial contact, pre-swing, and initial swing to control the prosthesis. In the control group, as shown in Figure 3, the 95 and 75% HDRs of muscle co-activation peaks predominately occur during stance, for both paces. During self-paced walking, shorter HDRs of muscle peaks also occur during pre-swing and initial swing. The HDRs are longer within the left limb than within the right limb, and present during the swing, regardless of gait speed, within the right limb. Gait asymmetry is observed among healthy intact individuals, however, causes are not fully understood. Some combination of asymmetries in morphology and neural processes likely contribute to asymmetrical gait measures (20). Sadeghi et al. (21) found that gait asymmetry reflects natural functional differences between limbs related to each limb's contribution to propulsion and control and varies by gait task. Additionally, asymmetry observed in this study might also be impacted by participants rounding corners (5 left and 1 right) during data collection. Because video was not a part of the protocol, it is unknown which strides occurred while rounding corners.

Compared with the controls' gait performance, individuals with OTFA experience co-contraction during relatively more of the gait cycle, especially within the intact limb. In the OTFA group, co-contraction in both limbs demonstrates diminished control of both limbs. Whereas, the control group demonstrated control bilaterally. For example, in Figure 3, the control group's most likely muscle co-activation peaks, in red or orange, are largely along or near the diagonal during periods of weight acceptance, and, with the right limb, weight transference. Some differences in co-contraction between participants with OTFA might be impacted by residuum length.

The high variance in co-activation time of the adductors, hamstrings, and quadriceps provide evidence of the contribution by distal-most residuum muscles to control the OTFA residuum within the prosthetic socket, similar to what was found in healthy men with OTTA (3). Higher variance in EMG activity and increased reliance on the intact limb is also consistent with results by Wentink et al. (11) from a heterogeneous TFA cohort and longer hip muscle activation times within intact and amputated limbs discovered by Jaegers et al. (12). However, these studies did not measure co-activation prior to averaging stride activation to verify co-activity of muscles during the same stride. These studies also report fewer than 24 strides per subject. The current study was able to obtain ≥67 strides per participant per trial and assesses the distributions of co-activation peak times between muscles during the same stride. Hong and Mun (4) report a significant relationship between when the hamstring-quadriceps activation occurred and the increase in RSI pressures. However, they did not report residuum adductor activation times and surgical approach type.

Typically, medial muscle co-contraction is positively correlated with the progression of medial knee osteoarthritis (OA) and lateral muscles co-contraction is negatively correlated with protection against exacerbating OA (22). Additionally, Schmitt et al. (23) found that increasing medial co-contraction led to an ineffective walking strategy that was harmful to joint integrity in individuals with unstable medial knee OA. Efficient use of distal residuum muscles during gait to reduce contraction within the intact limb may improve OTFA outcomes. Moreover, the current study's results may be unique to the osteomyoplastic surgical approach. Future comparison of distal-most residuum muscle activation during prosthetic gait between osteomyoplastic and traditional approaches is warranted, as these result in distinct residuum musculature.

This study examined muscle co-activation of the hip adductors, hamstrings, and quadriceps within the OTFA residuum concomitantly with the contralateral intact limb. Furthermore, this study examined distal-most residuum muscle activation and RSI pressures during brisk and self-paced gait in a homogeneous OTFA cohort. However, results should not be considered conclusive, as there were only six participants with varying residuum lengths and available residuum muscle mass. Results may vary, due to these potentially confounding factors. However, the results will inform larger, future work to confirm the study's results. Additionally, the OU-PAM used to collect gait data has yet to be validated in the TFA study population and does not instrument the foot pressures of the OTFA cohort. Future studies comparing the OU-PAM with 3D motion and force plate capture concomitantly with EMG would be ideal to provide instrument validity.

## Conclusion

In a cohort of six, otherwise-healthy men with unilateral OTFA, hip adductor-hamstrings-quadriceps co-contraction occurred during weight transfer from one limb to the other, in both intact and residual limbs during brisk and self-paced walking. For the remainder of the gait cycle, co-contraction also occurs within the intact limb. The results indicate that rehabilitation professionals should consider prescribing specific therapeutic exercises as part of OTFA rehabilitation to improve controlled activation of hip adductors, hamstrings, and quadriceps in the intact and distal-residual limbs to improve overall gait performance stability and reduce excessive energy expenditure. Furthermore, individuals with OTFA consciously and actively employ the distal muscles during bodyweight transfer between limbs during prosthetic gait training, early in rehabilitation, and throughout long-term follow-up, to help maintain gait performance stability and minimize risk of falls.

## Data Availability Statement

The raw data supporting the conclusions of this article will be made available by the authors, without undue reservation.

## Ethics Statement

The studies involving human participants were reviewed and approved by University of Oklahoma Health Sciences Center Institutional Review Board. The patients/participants provided their written informed consent to participate in this study.

## Author Contributions

We performed the osteomyoplastic amputation of all participants in the study with amputation. JD designed the prostheses of all participants in the study with amputation. CD was the PI of the overall study. KV, JD, WE, and CD contributed to the conception and design of the study. KV, JD, CD, AF, and MS were involved in gait data acquisition. MS performed the data processing and analysis and wrote the first draft of the manuscript. CD and AF wrote sections of the manuscript. All authors contributed to manuscript revision, read, and approved the submitted version.

## Funding

The overall project was funded in part by the Oklahoma Center for the Advancement of Science and Technology Health Research Program (HR11-097).

## Conflict of Interest

The authors declare that the research was conducted in the absence of any commercial or financial relationships that could be construed as a potential conflict of interest.

## Publisher's Note

All claims expressed in this article are solely those of the authors and do not necessarily represent those of their affiliated organizations, or those of the publisher, the editors and the reviewers. Any product that may be evaluated in this article, or claim that may be made by its manufacturer, is not guaranteed or endorsed by the publisher.

## Supplementary Material

Supplementary Figure S1 Sensor Placement [Shotande et al. (10), submitted]. Pressure and muscle activation data are simultaneously collected using the OU-PAM (3, 14), which can capture up to 16 analog input channels at 1 kHz. It utilizes the STK525 (15) and ATEVK525 (15) boards for data acquisition. Differences in sensor placements between the osteomyoplastic transtibial amputation (OTTA) cohort in the study done by Mai et al. (3) and the osteomyoplastic transtibial amputation (OTTA) cohort in this paper: (1) two residuum electromyography (EMG) sensors are moved from the tibialis anterior and gastrocnemuis for the OTTA cohort to the adductors and hamstrings for the OTFA cohort (quadriceps are recorded for both cohorts), and (2) residuumÂ-socket interface (RSI) pressures are collected at the distal, middle and proximal levels for individuals with OTTA and only the distal and proximal levels for individuals with OTFA, **(A)** two rings of pressure sensors are placed within the residuum socket at the 1/3-femur length and distal femur levels. At each level, sensors were placed at the anterior, posterior, medial, and lateral positions, and **(B)** EMG sensors were placed on the respective external muscle bellies of the quadriceps, hamstrings, and adductor muscles.

Supplementary Figure S2 Experiment protocol diagram [Dionne et al. (1)]. Analysis in this study focused on data collected during brisk and self-paced walking.

Supplementary Figure S3 Distributions of stance duration in time (seconds) and as a fraction of the gait cycle for OTFA and control cohorts during brisk (blue) and self-paced (magenta) walking, from the best visit. The vertical dashed lines indicate the mode of the distributions. The horizontal red dashed lines indicate the probability cut-off for the 95% high density regions (HDRs). The green horizontal lines with diamond endpoints indicate the HDRs. Mo is short for the mode of the corresponding distribution, and n indicates the number of strides extracted. The distributions of stance duration are consistently narrower and start later for the control group compared to the OTFA group, as evidenced by the HDRs.

Supplementary Figure S4 Modes of residuum-socket interface (RSI) pressure **(A)** and electromyography (EMG) activation **(B)** peak times from 6 individuals with OTFA. Rows are individual participants. **(A)** The most likely RSI pressure peaks occur prior to 60% of the gait cycle, for both walking speeds. **(B)** EMG activation modes are distributed throughout the gait cycle, indicating a high variance in peak time. Vertical clusters where modes between subjects approximately line up, indicate common muscle recruitment strategies between individuals.

Supplementary Figure S5 Modes of foot sensor pressure **(A)** and EMG activation **(B)** peak times from 6 matched controls. Rows are individual participants. Vertical clusters where modes between subjects approximately line up, indicate common heel-strike or push-off times and muscle recruitment strategies between individuals. **(A)** The most likely toe pressure peaks are at 50%. The most likely heel pressure peaks are at 10 and 30%.

Supplementary Figure S6 OTFA muscle EMG matched peaks [Shotande et al. (10), submitted]. Each point corresponds to a single stride. Time zero corresponds to the heel strike of the corresponding limb. Colors indicate a specific subject. Points along the diagonal indicate co-activation of the muscle pair during the stride.

Supplementary Figure S7 Controls matched peaks. Each point corresponds to a single stride. Time zero corresponds to the heel strike of the corresponding limb. Colors indicate a specific subject. Points along the diagonal indicate co-activation of the muscle pair.

Supplementary Table S1 Results of the KS tests, for the individuals with osteomyoplastic transfemoral amputation (OTFA), comparing the shape of the distributions of peak times between brisk and self-paced walking for each pressure location **(A)** and muscle group **(B)**. α= 0.05 is corrected to 0.0064 for **(A)** and to 0.0085 for **(B)** to account for multiple comparisons (16). Statistically significant differences are indicated by the *.

Supplementary Table S2 Results of the KS tests, for the matched controls, comparing the shape of the distribution of peak times between brisk and self-paced walking for each pressure location **(A)** and muscle group **(B)**. α= 0.05 is corrected to 0.013 for **(A)** and to 0.0085 for **(B)** to account for multiple comparisons (16). Statistically significant differences are indicated by the *.
