# Supplementary figures and images for: Comparing Temporospatial Performance During Brisk and Self-Paced Walking by Men With Osteomyoplastic Transfemoral Amputation and Controls Using Pressure and Muscle Activation Peak Times
Source: Front Rehabil Sci. 2022 May 6;3:848657. doi: 10.3389/fresc.2022.848657 (PMC9397685; doi:10.3389/fresc.2022.848657)

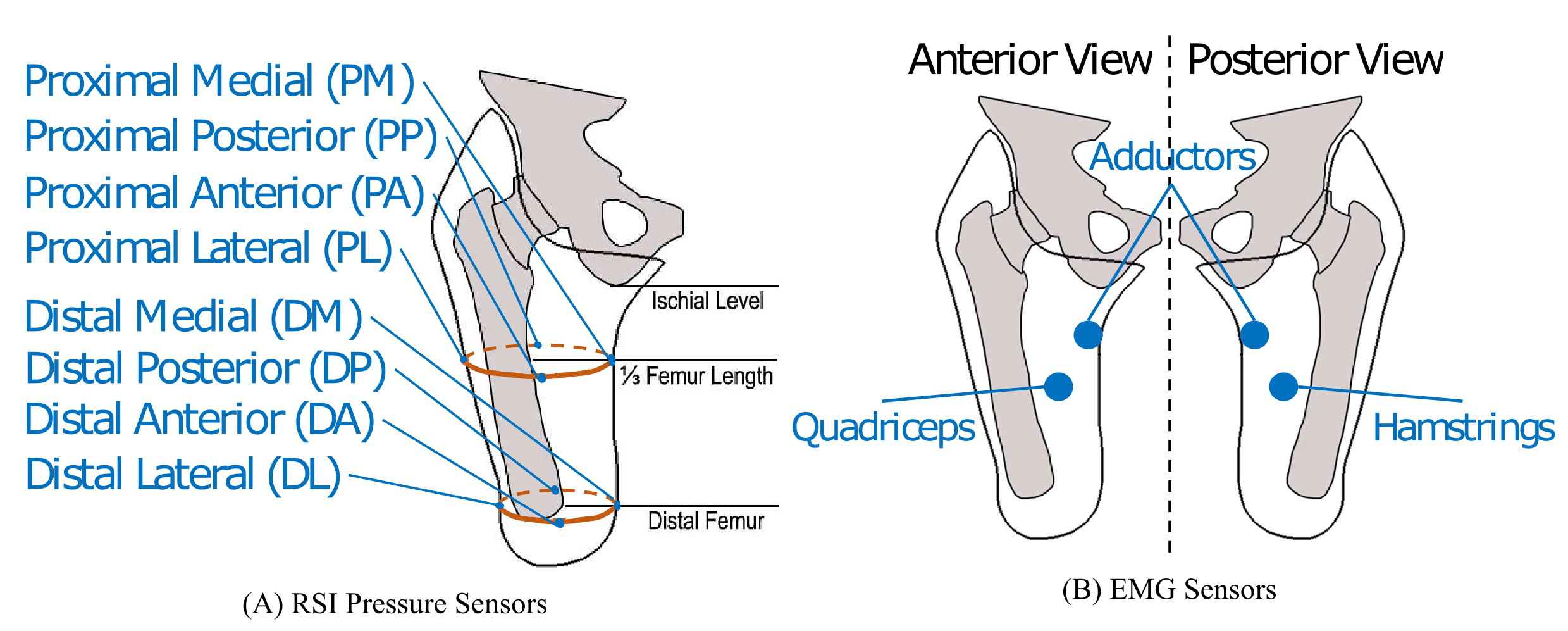

Supplement: Supplementary Figure S1 — Sensor Placement [Shotande et al. (10), submitted]. Pressure and muscle activation data are simultaneously collected using the OU-PAM (3, 14), which can capture up to 16 analog input channels at 1 kHz. It utilizes the STK525 (15) and ATEVK525 (15) boards for data acquisition. Differences in sensor placements between the osteomyoplastic transtibial amputation (OTTA) cohort in the study done by Mai et al. (3) and the osteomyoplastic transtibial amputation (OTTA) cohort in this paper: (1) two residuum electromyography (EMG) sensors are moved from the tibialis anterior and gastrocnemuis for the OTTA cohort to the adductors and hamstrings for the OTFA cohort (quadriceps are recorded for both cohorts), and (2) residuumÂ-socket interface (RSI) pressures are collected at the distal, middle and proximal levels for individuals with OTTA and only the distal and proximal levels for individuals with OTFA, (A) two rings of pressure sensors are placed within the residuum socket at the 1/3-femur length and distal femur levels. At each level, sensors were placed at the anterior, posterior, medial, and lateral positions, and (B) EMG sensors were placed on the respective external muscle bellies of the quadriceps, hamstrings, and adductor muscles. [file Image_1.JPEG]

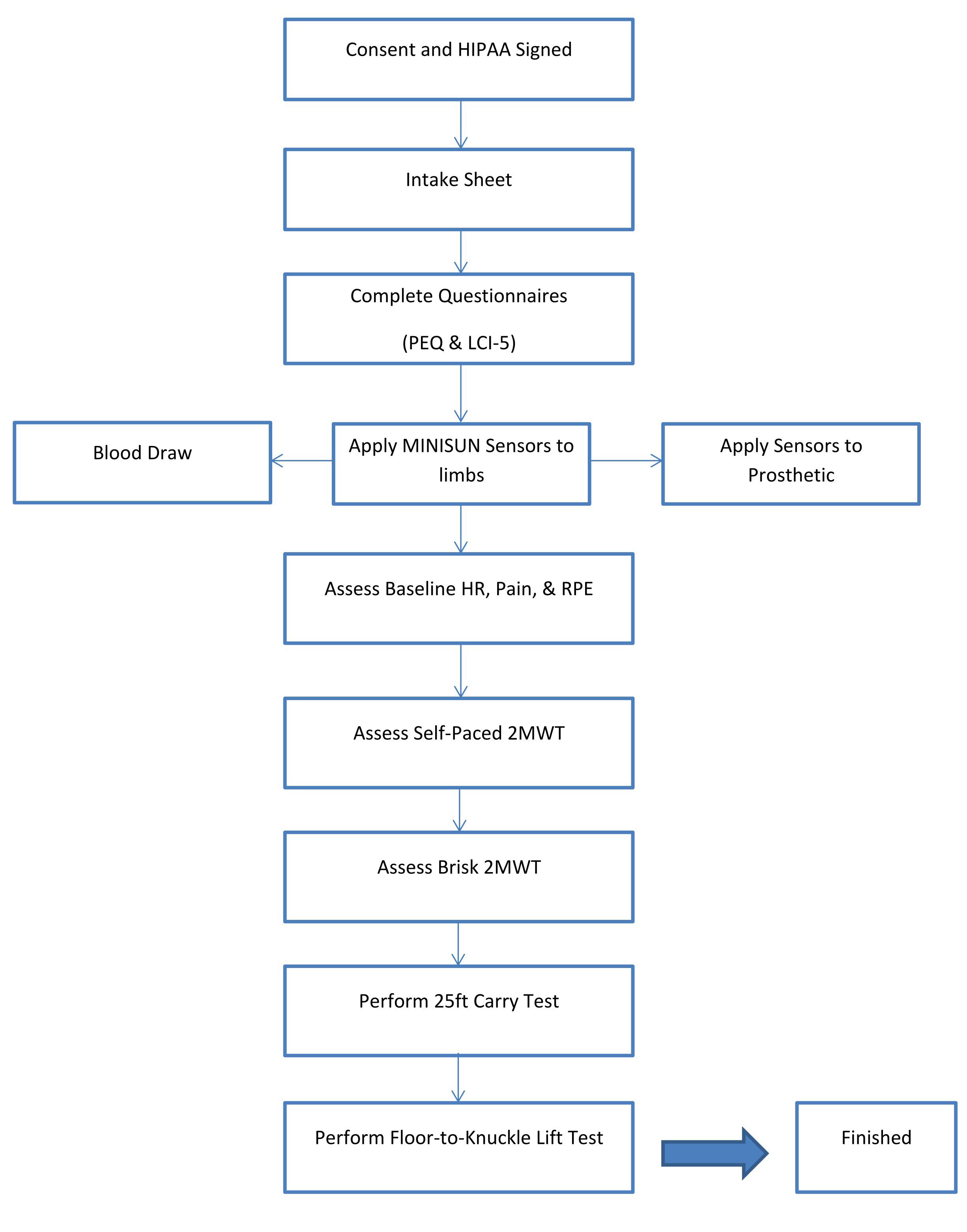

Supplement: Supplementary Figure S2 — Experiment protocol diagram [Dionne et al. (1)]. Analysis in this study focused on data collected during brisk and self-paced walking. [file Image_2.JPEG]

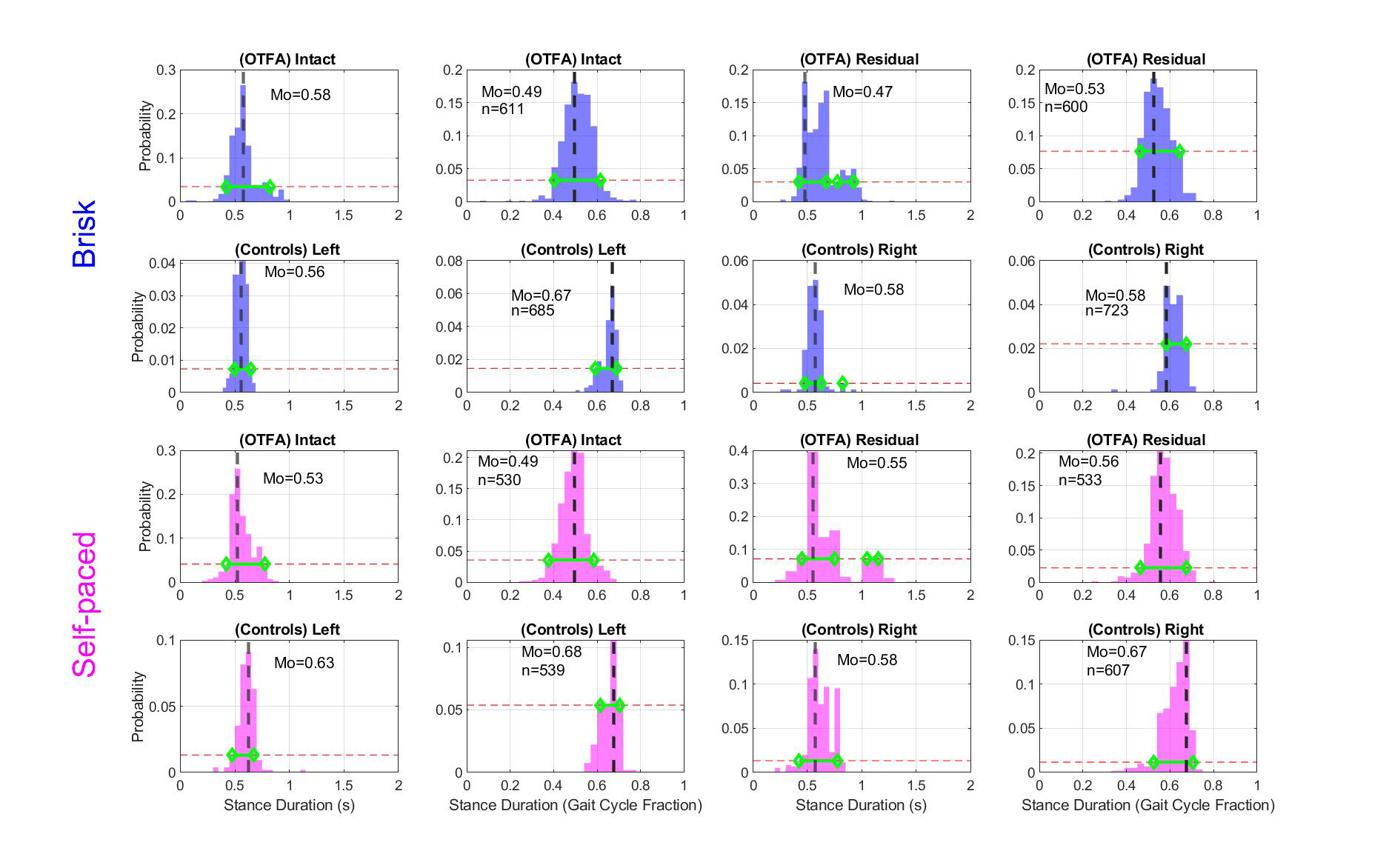

Supplement: Supplementary Figure S3 — Distributions of stance duration in time (seconds) and as a fraction of the gait cycle for OTFA and control cohorts during brisk (blue) and self-paced (magenta) walking, from the best visit. The vertical dashed lines indicate the mode of the distributions. The horizontal red dashed lines indicate the probability cut-off for the 95% high density regions (HDRs). The green horizontal lines with diamond endpoints indicate the HDRs. Mo is short for the mode of the corresponding distribution, and n indicates the number of strides extracted. The distributions of stance duration are consistently narrower and start later for the control group compared to the OTFA group, as evidenced by the HDRs. [file Image_3.JPEG]

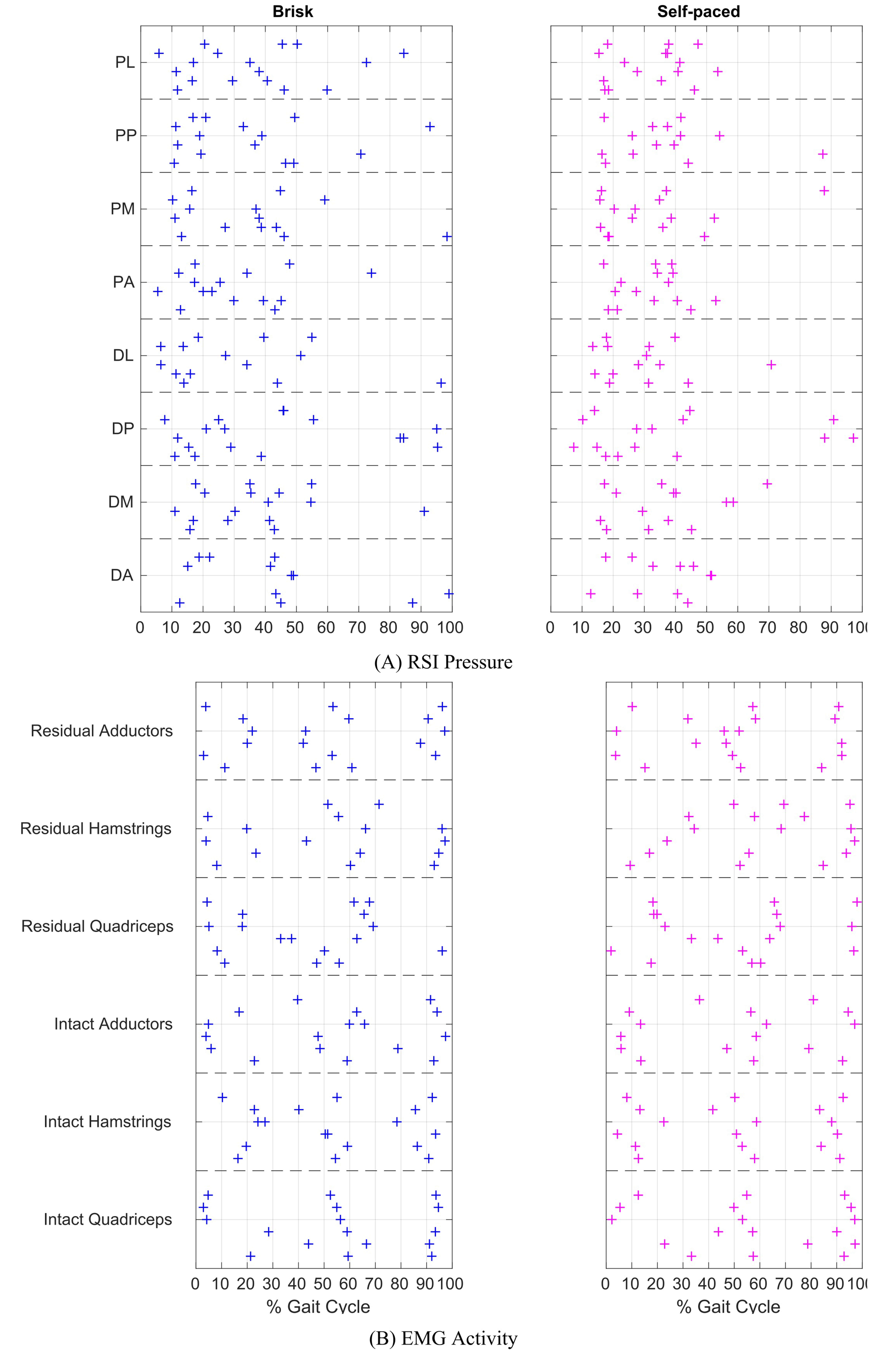

Supplement: Supplementary Figure S4 — Modes of residuum-socket interface (RSI) pressure (A) and electromyography (EMG) activation (B) peak times from 6 individuals with OTFA. Rows are individual participants. (A) The most likely RSI pressure peaks occur prior to 60% of the gait cycle, for both walking speeds. (B) EMG activation modes are distributed throughout the gait cycle, indicating a high variance in peak time. Vertical clusters where modes between subjects approximately line up, indicate common muscle recruitment strategies between individuals. [file Image_4.JPEG]

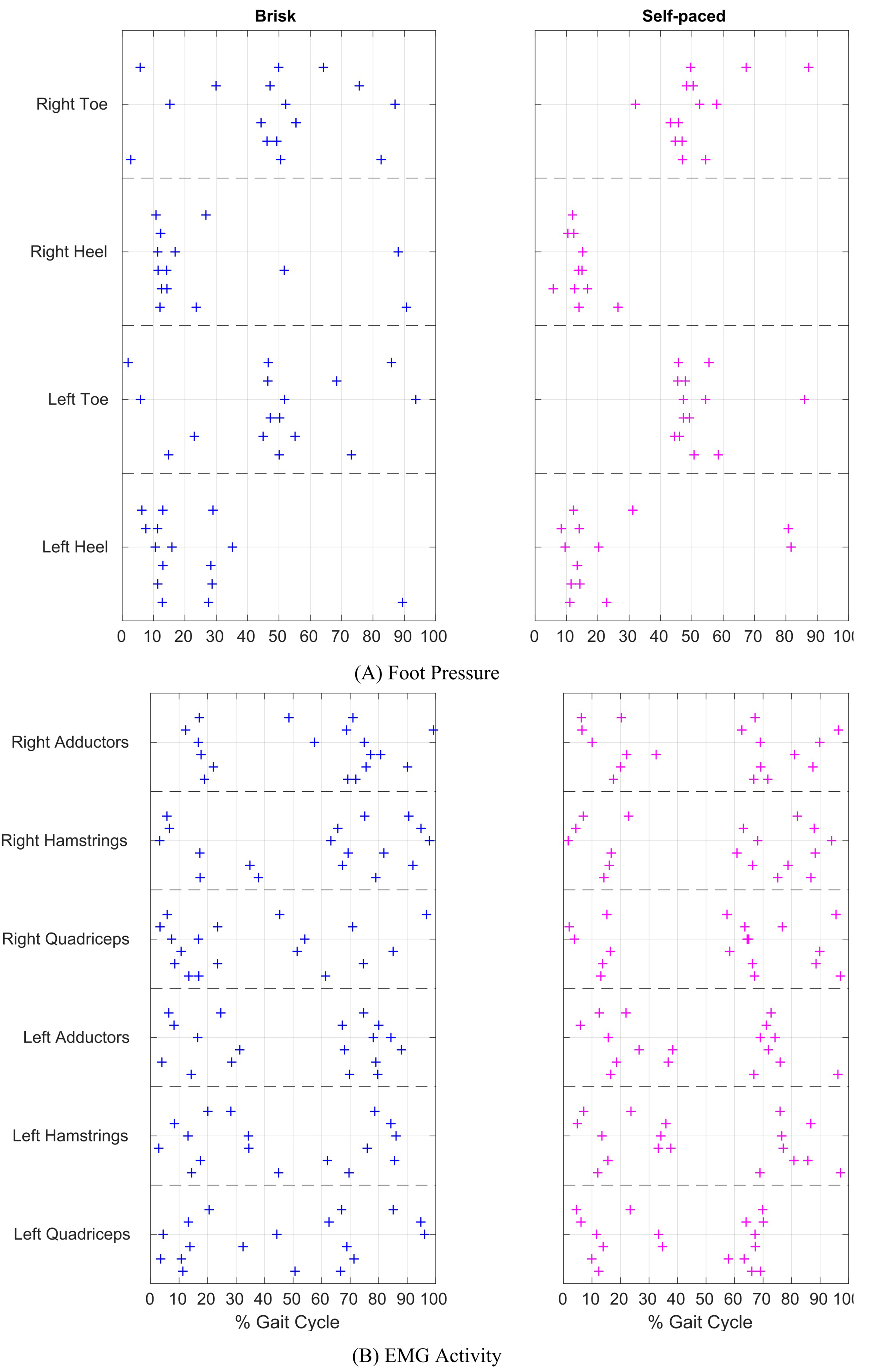

Supplement: Supplementary Figure S5 — Modes of foot sensor pressure (A) and EMG activation (B) peak times from 6 matched controls. Rows are individual participants. Vertical clusters where modes between subjects approximately line up, indicate common heel-strike or push-off times and muscle recruitment strategies between individuals. (A) The most likely toe pressure peaks are at 50%. The most likely heel pressure peaks are at 10 and 30%. [file Image_5.JPEG]

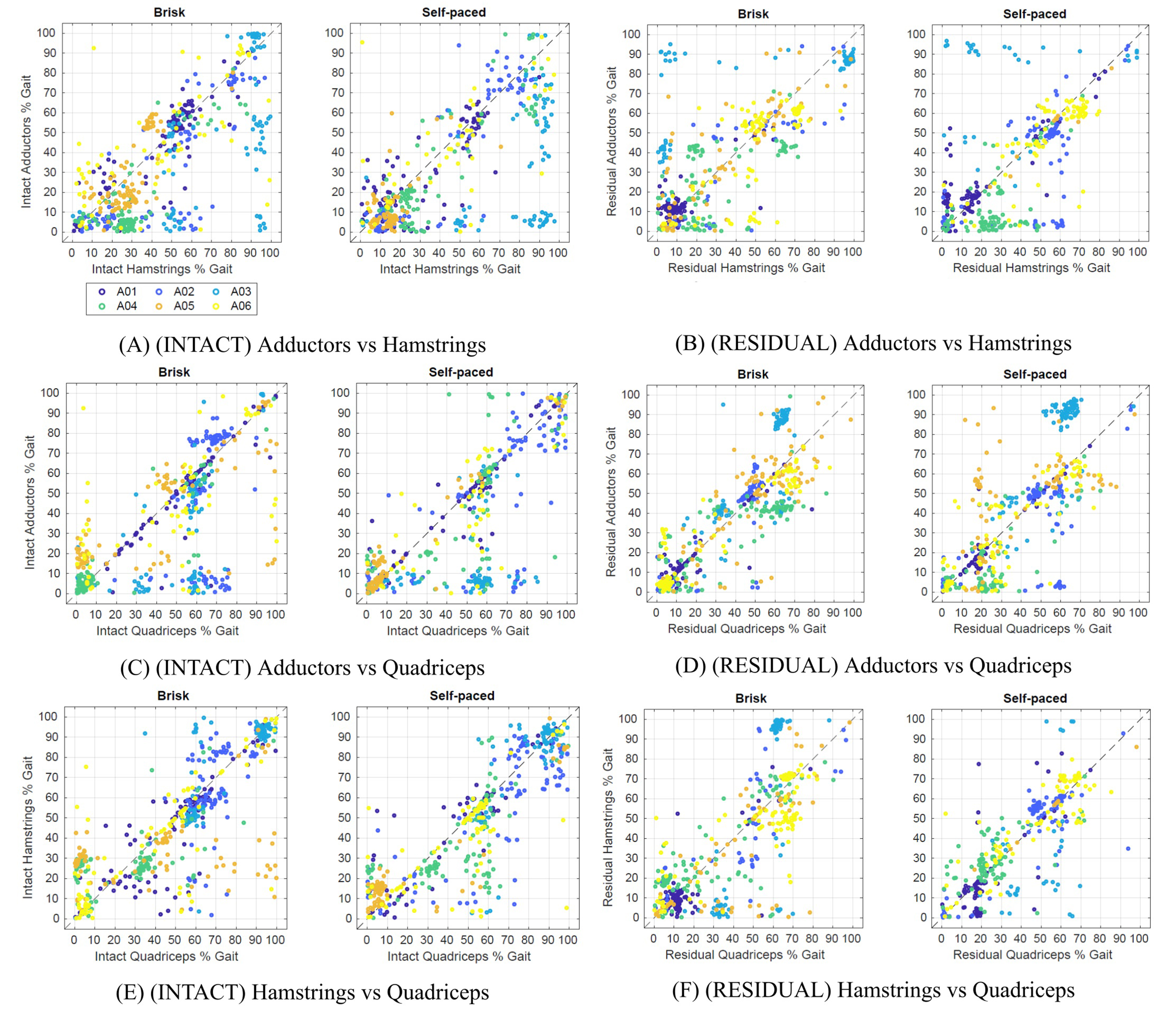

Supplement: Supplementary Figure S6 — OTFA muscle EMG matched peaks [Shotande et al. (10), submitted]. Each point corresponds to a single stride. Time zero corresponds to the heel strike of the corresponding limb. Colors indicate a specific subject. Points along the diagonal indicate co-activation of the muscle pair during the stride. [file Image_6.JPEG]

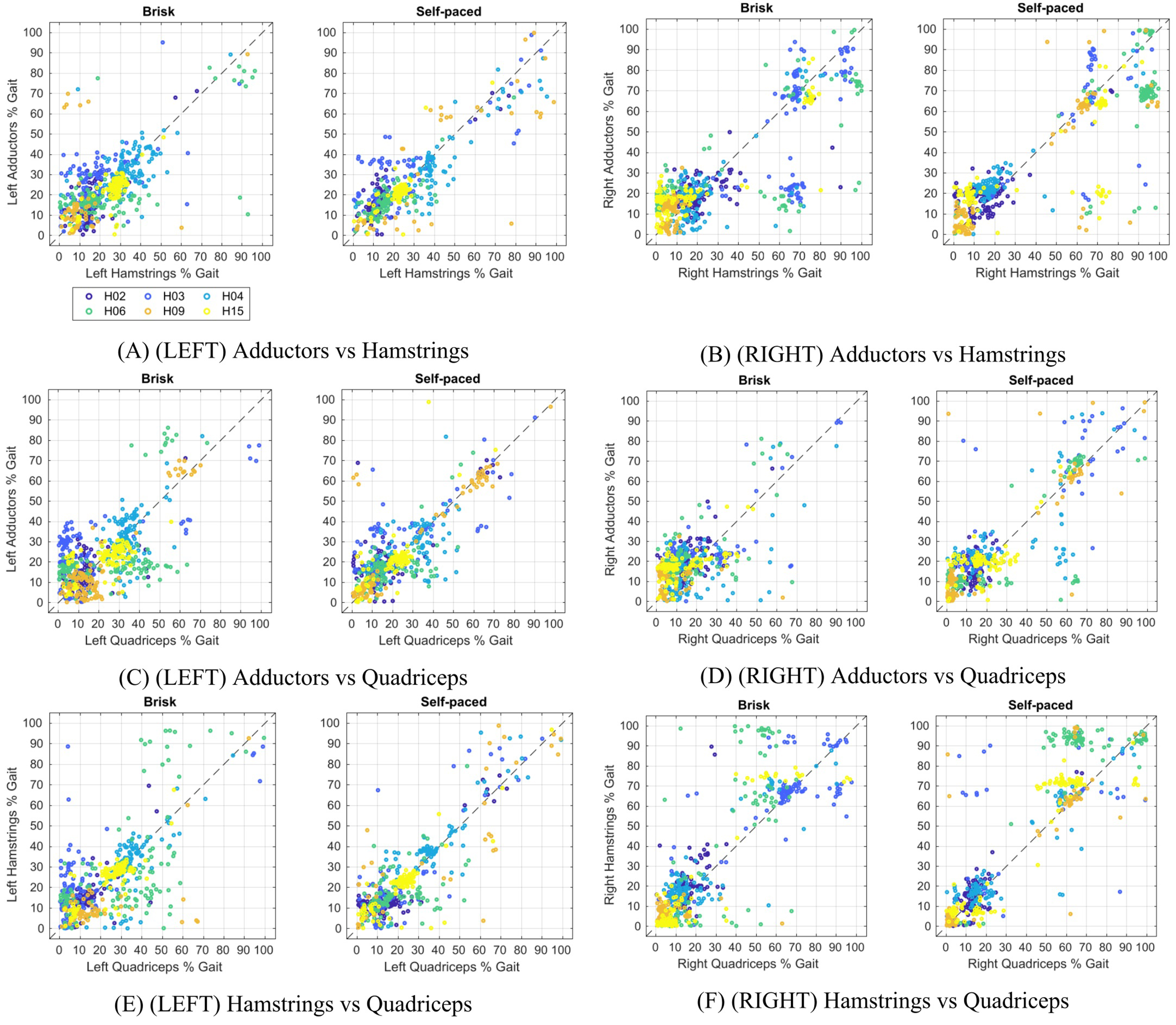

Supplement: Supplementary Figure S7 — Controls matched peaks. Each point corresponds to a single stride. Time zero corresponds to the heel strike of the corresponding limb. Colors indicate a specific subject. Points along the diagonal indicate co-activation of the muscle pair. [file Image_7.JPEG]
